# RGO-Pt as an effective catalyst for U(IV) generation under hydrogen

**DOI:** 10.1038/s41598-025-08442-z

**Published:** 2025-07-07

**Authors:** Kuntal Kumar Pal, Ramakrishna Reddy, Chanchal Ghosh, Sandip Dhara

**Affiliations:** 1 Reprocessing Material Development Section, Process Radiochemistry Reprocessing Research and Development Division, Reprocessing Group, Indira Gandhi Centre for Atomic Research, Kalpakkam, 603102 India; 2 Indira Gandhi Centre for Atomic Research, A CI of Homi Bhabha National Institute, Kalpakkam, 603102 India; 3 Minor Actinide Chemistry and Reconversion Section, Process Radiochemistry Reprocessing Research and Development Division, Reprocessing Group, Indira Gandhi Centre for Atomic Research, Kalpakkam, 603102 India; 4 Physical Metallurgy Division, Metallurgy and Materials Group, Indira Gandhi Centre for Atomic Research, Kalpakkam, 603102 India; 5 Material Science Group, Indira Gandhi Centre for Atomic Research, Kalpakkam, 603102 India

**Keywords:** RGO-Pt, Catalysis, Uranium reduction, Hydrogenation, Catalysis, Energy, Materials chemistry, Nuclear chemistry

## Abstract

Effective generation of U(IV) from U(VI) is a key step to achieve the partitioning of Pu from U in a spent nuclear fuel reprocessing plant. Reduction under a hydrogen atmosphere is one of the methods for the generation of U(IV) from U(VI) in the presence of a catalyst. Towards this, we have developed novel reduced graphene oxide (RGO)-supported Pt catalysts with varying RGO to Pt ratios. All these catalysts were tested for their catalytic performance for U(VI) to U(IV) reduction under hydrogen, and the performance was compared with the literature-reported SiO_2_-Pt model catalyst. During the catalytic study, the dual role of Pt was confirmed. It helps the dispersion of materials in the aqueous reaction medium and provides the active surface for the U(VI) reduction reaction using hydrogen. In this regard, Pt nanoclusters with larger sizes were identified for effective catalysis instead of the smallest size.

**Supplementary Information:**

The online version contains supplementary material available at 10.1038/s41598-025-08442-z.

## Introduction

Plutonium, a manmade actinide element that has attracted much attention in the past decades, is produced by the neutron capture by fertile U-238, followed by successive β decay in a nuclear reactor. Apart from its strategic importance, in nuclear energy Pu is particularly important for its use as a U–Pu mixed oxide (MOX) fuel in fast reactors^1,2^. Moreover, Pu, being highly radiotoxic, separation of Pu from spent fuel and further use as a fissile element in a reactor drastically reduces the radiotoxicity of the spent fuel, along with the volume reduction of the radioactive waste discharge from the reactors. In particular, the radiotoxicity of the spent fuel can be reduced to natural uranium background in a time span of 10,000 years from 300,000 years by separating U and Pu from the spent nuclear fuel. Further reduction of the time span to a reasonable period of around 300 years is also possible by partitioning and transmutation (P&T) of the remaining long half-life radionuclides from the spent fuel^3^.

However, much of this Pu, which comes from the spent nuclear fuel, needs extensive separation and purification from the rest of the fission products and actinides before being used as fresh fuel for a reactor. So far, the plutonium uranium reduction extraction (PUREX) process has been extensively adopted in various reprocessing plants worldwide to recover the U and Pu together from spent nuclear fuel^4,5^. PUREX needs an additional partitioning step to separate the Pu from the U and Pu mixed phase. The separation is generally achieved by reducing the Pu(IV) to its in-extractable Pu(III) state using a suitable reducing agent. Historically, ferrous sulfamate^6^, hydroxylamine nitrate^7,8^, a combination of both^9^, and acetohydroxamic acid^10^ were used in the partitioning step to achieve the selective reduction of Pu. However, later, all these reagents were withdrawn due to their associated problems; ferrous sulphamate adds additional salt and corrosive sulphate ions to the waste stream, creating issues with associated waste management, hydroxylamine nitrate possesses poor reduction kinetics and is suitable only for lower nitric acid conditions, which is not the case in commercial reprocessing plants. Later, all these reagents are replaced exclusively by uranous nitrate (U(IV)-nitrate), which is presently being used in commercial reprocessing plants due to its added advantages, such as no addition of any foreign salt into the process stream, stability of U(IV) in both aqueous and organic phase and thus achieving reduction in both aqueous and organic phase^5,6^. Hence, it is essential to efficiently produce U(IV) in a fast reactor fuel reprocessing plant, where a large quantity of Pu is reprocessed.

So far, due to its ease of operation and reliability, the electrochemically produced U(IV) has been used extensively for thermal reactor fuel reprocessing with a limited Pu load (0.3%)^11,12^. However, disadvantages like poor reaction kinetics, less efficiency (60 to 70%), and high overpotential restrict the use of electrochemical methods for the bulk-scale efficient production of U(IV) for processing large quantities of Pu from fast reactor fuel. Towards this, people have studied the supported Pt like Pt/SiO_2_^13–15^, Pt/Al_2_O_3_^16^ and PtO_2_^17^ as catalysts for the catalytic reduction of U(VI) to U(IV). So far, inorganic support materials have mostly been used to synthesize the Pt-loaded catalysts for U(VI) reduction purposes. However, since these catalysts are used in a radioactive solution, the safe disposal of these catalysts also needs to be addressed before their bulk-scale production and implementation in a commercial fast reactor fuel reprocessing plant. With the present design of these catalysts, it is not possible to reduce their volume by incineration, the most commonly used practice for waste volume reduction in the industry.

Graphene and related materials have drawn much of the attention of the scientific community in the recent past due to their remarkable chemical and physical properties. However, the bulk-scale production of ideal graphene nano-sheets remains a challenge, and even if it is produced, powder processing and aqueous processing of this material are not possible with the presently available technology^18^. Towards this, the closest available materials are graphene oxide (GO) and reduced graphene oxide (RGO), with the possibility of bulk-scale production and aqueous/solution processing. These materials are made up of carbon and oxygen and are completely incinerable. Moreover, due to the very high surface area of these materials as compared to presently used inorganic support materials, one can load a higher amount of active catalyst material (Pt) without compromising the Pt utilization efficiency and thus greatly reduce the total volume of the catalyst materials to be used for the reduction purpose; to ultimately reduce the volume of the waste catalyst by many folds.

With the above motive, we have loaded RGO with different proportions of Pt to reduce U(VI) to U(IV) in a plant-suitable acidic solution under a hydrogen atmosphere. Studies have also been performed to evaluate the role of RGO in catalysis. We have also studied the role of Pt in dispersing the RGO-Pt materials in the aqueous nitric acid reaction solution. The results of these studies are reported here.

## Materials and methods

### Materials

The chemicals used for the experiment were procured from the following vendors.

Graphite powder, chloroplatinic acid hexahydrate (H_2_PtCl_6_.6H_2_O) and ferroin indicator were procured from Sigma-Aldrich. Sulphuric acid (H_2_SO_4_), nitric acid (HNO_3_), hydrochloric acid (HCl) and hydrazine hydrate (N_2_H_4_.H_2_O) were procured from Merck. Orthophosphoric acid (H_3_PO_4_) was procured from Rankem Chemicals. Potassium permanganate (KMnO_4_) was procured from Alfa Aesar. Ethylene glycol (EG) was procured from Sisco Research Laboratories Pvt. Ltd. Ethanol was procured from Hayman Ltd. All these chemicals were of analytical grade and used directly without further purification. Uranium used for the experiments was sourced from the Nuclear Fuel Complex, Hyderabad, India, in the form of a UO_2_ pellet and used after dissolving it in concentrated HNO_3_. Hydrogen gas (Purity: 99.99%) used in the experiments was supplied by Atmospheric Speciality Gases Private Limited, Ahmedabad, India.

Deionized water obtained from a Millipore water system was used for all the experiments.

### Synthesis

#### Synthesis of graphene oxide (GO)

GO was synthesized directly from graphite powder via the improved Hummers’ method^19^. In a typical synthesis, 2 g of graphite powder was taken in a 500 mL beaker with 12 g of KMnO_4_ and mixed homogeneously with a glass rod. In another beaker, 26 mL of H_3_PO_4_ was mixed with 240 mL of concentrated H_2_SO_4_ and kept in an ice bath for 15 min. Finally, an ice-cooled acid mixture was added to the graphite and KMnO_4_ mixture. During the addition, the temperature of the reaction mixture was maintained well below room temperature to avoid any high-temperature oxidation of the graphite powder. Finally, the suspension was kept in an oil bath at 50 °C for 12 h. under continuous stirring. After 12 h, the completed reaction mixture was cooled to room temperature and slowly poured into 300 mL ice-cooled water with 5 mL of H_2_O_2_. Finally, the yellow solid suspension was separated from the rest of the solution via centrifugation and washed several times with 32% HCl, followed by ethanol. The product was dried overnight in a vacuum oven at 75 °C.

#### Synthesis of RGO-Pt

For the synthesis of Pt-decorated RGO, the in-situ polyol reduction method was adopted, as reported by Yongjie Li et al*.*^20^, with slight modification. In brief, for 10% Pt loading, 180 mg of GO was dispersed into an 80 mL ethylene glycol and 18.6 mL water mixture in an ultrasonic bath for 6 h. A solution of 1.4 mL 74 mM chloroplatinic acid was added to the GO dispersion and mixed thoroughly for 30 min. Finally, the solution was transferred to a 200 mL Teflon-lined hydrothermal autoclave. The autoclave was transferred to a hot air oven and heated at 120 °C for 12 h. Finally, the Pt-loaded reduced graphene oxide (RGO-Pt) was separated from the solution by centrifugation and washed several times with water, followed by ethanol.

For the synthesis of 5% and 2.5% Pt-loaded RGO-Pt, the GO and H_2_PtCl_6_ solution amount was adjusted accordingly, where 80% EG-water medium was maintained for all the syntheses of RGO-Pt.

#### Synthesis of SiO_2_-Pt model catalysts

The SiO_2_-Pt model catalysts were synthesized as per the previous report^21^ using the wet impregnation method. In brief, SiO_2_ powder was initially dispersed in water. Followed by, the required amount of H_2_PtCl_6_ solution (Equivalent to 0.85 wt.%) was added to the dispersion and the mixture was stirred for 24 h. Finally, the water was evaporated in a rotary evaporator, and the dry powder was subject to reductive heat treatment at 300 °C in a tubular furnace under 8% H_2_-Ar atmosphere in order to obtain the SiO_2_-Pt model catalysts.

### Characterization techniques

Pt content in all RGO-Pt samples was determined using inductively coupled plasma optical emission spectroscopy (ICP-OES; Spectro Arcos, Germany). In a typical analysis, a known weight of the sample was dissolved in a known volume of aqua regia and this solution was subjected to ICP-OES analysis. The phase identification in all these samples was performed using powder X-ray diffraction (XRD) with Co- Kα radiation source. Raman spectra were recorded using the Renishaw Invia Raman microscope to understand the structural properties of graphene materials. Attenuated total reflectance Fourier transform infrared (ATR-FTIR) spectra of all the samples were recorded using a BRUKER ALPHA II spectrophotometer to get information about the functional groups present in the graphene materials. FESEM images were recorded using a Zeiss Ultra Plus FESEM microscope. High-resolution transmission microscopy (HRTEM) and high-angle annular dark-field (HAADF) images were recorded in a M/s. ThermoFisher Scientific make Talos 200FS TEM. Elemental mapping data were recorded using 2 quadrant Energy-dispersive X-ray spectroscopy (XEDS) detector. UV–Vis spectra were recorded using Thermo Scientific Evolution One Plus UV–Vis spectrophotometer. Total organic carbon (TOC) content in the samples was measured using Analytic Jena multi N/C 2100S TOC analyser.

### Uranium reduction studies

Uranium reduction studies were carried out in a 0.25 M U(VI) solution. HNO_3_ and hydrazine concentrations were maintained in the reaction medium at 1.3 M and 0.2 M, respectively. Two strategies were followed for catalyst addition. One strategy was the direct addition of the catalyst in a 100 mL batch of U(VI), which was taken in a 450 mL autoclave. In other cases, catalysts were added to the U(VI) solution well before the start of the reduction reaction and put in an ultrasonic bath for 3 h for dispersion. A hydrogen pressure of 2 bar was maintained inside the reactor, and the solution was stirred with a motorized external stirrer at 1200 rpm.

**Fig. 1:**
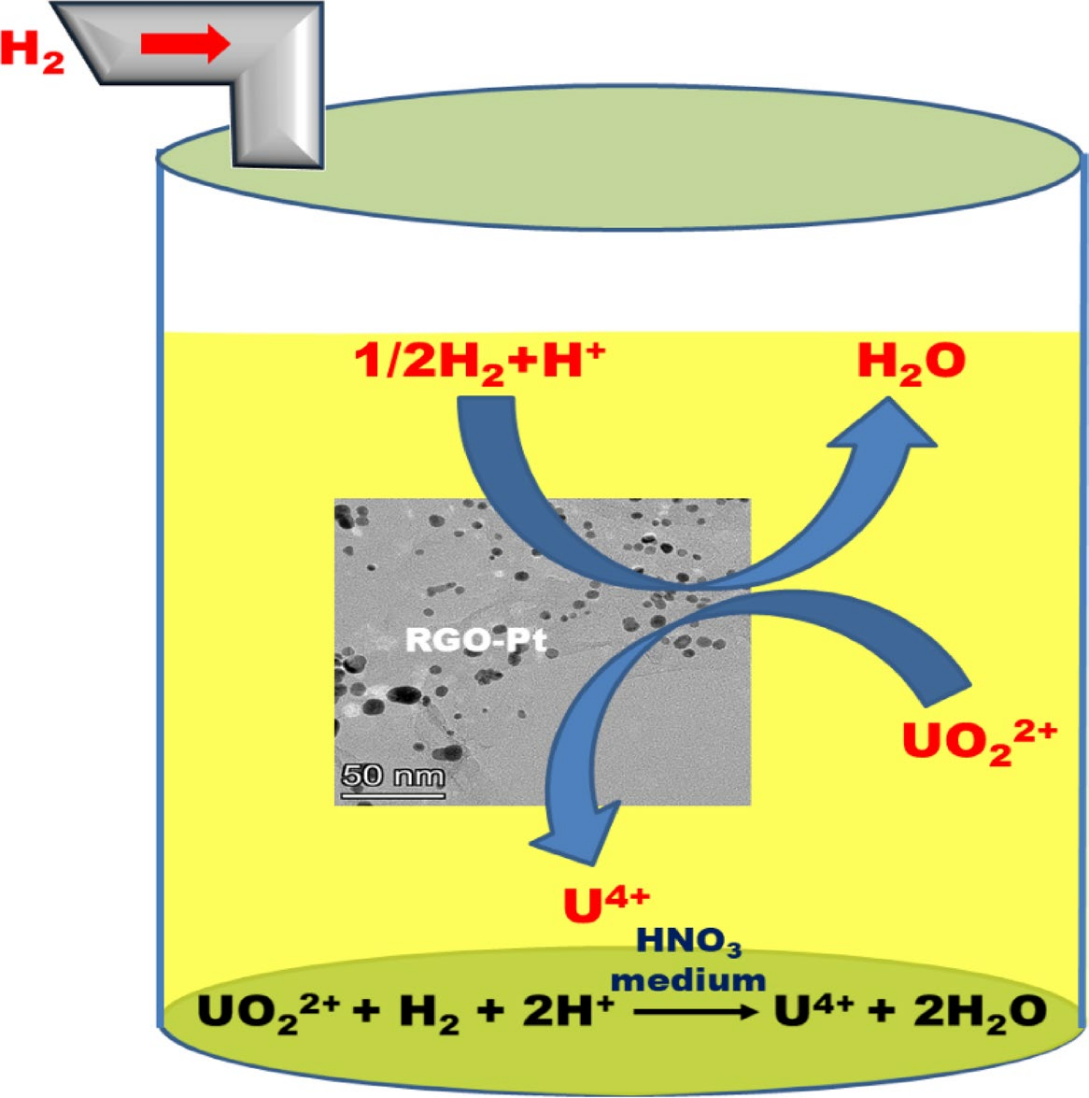
Schematic of the reduction of U(VI) to U(IV) over RGO-Pt using hydrogen.

Liquid samples were collected at regular fixed time intervals. After centrifugation, a desired amount of the aliquot was taken and titrated against potassium dichromate in ~ 2 (M) H_2_SO_4_ medium in the presence of ferroin indicator. For all the experiments involving RGO-Pt catalysts, a Pt catalyst to U atomic ratio was maintained at 1:5000. Figure 1 shows the schematic of the reaction inside the reactor.

## Results and discussion

### Synthesis and characterization

The improved Hummers method was chosen as a path for the synthesis due to its several advantages over other popular methods like the Hummers’ and modified Hummers’ method. The advantages of the improved Hummers’ method, as reported by Daniela et al.^19^, are higher oxidation efficiency, leading to high product yield, no toxic gas evolution during the synthesis and an easy-to-adopt methodology, leading to its scalability. The method also retains a greater amount of basal plane framework in the GO than the other methods for GO synthesis^19^. This retained framework may help decorate the Pt nanoparticle over the basal plane of the material in the latter stage and also help maintain good electrical conductivity in the final material.

For the decoration of Pt, the in-situ polyol reduction method, as reported by Yongjie Li et al*.*^20^, was modified suitably to enable the large-scale synthesis of the catalyst material in a safer way. In our case, we followed the incubation of the reaction mixture at an elevated temperature in a closed Teflon-lined stainless-steel container for 12 h without any continuous stirring to obtain the catalyst material. This method drastically improves the safety and scalability of this synthetic method with no direct human interference during the course of the reaction.

The final amount of the Pt present in the catalysts, as estimated by ICP-OES analysis, is 2.58% for RGO-Pt (2.5%), 5.8% for RGO-Pt (5%) and 10.86% for RGO-Pt (10%).

**Fig. 2:**
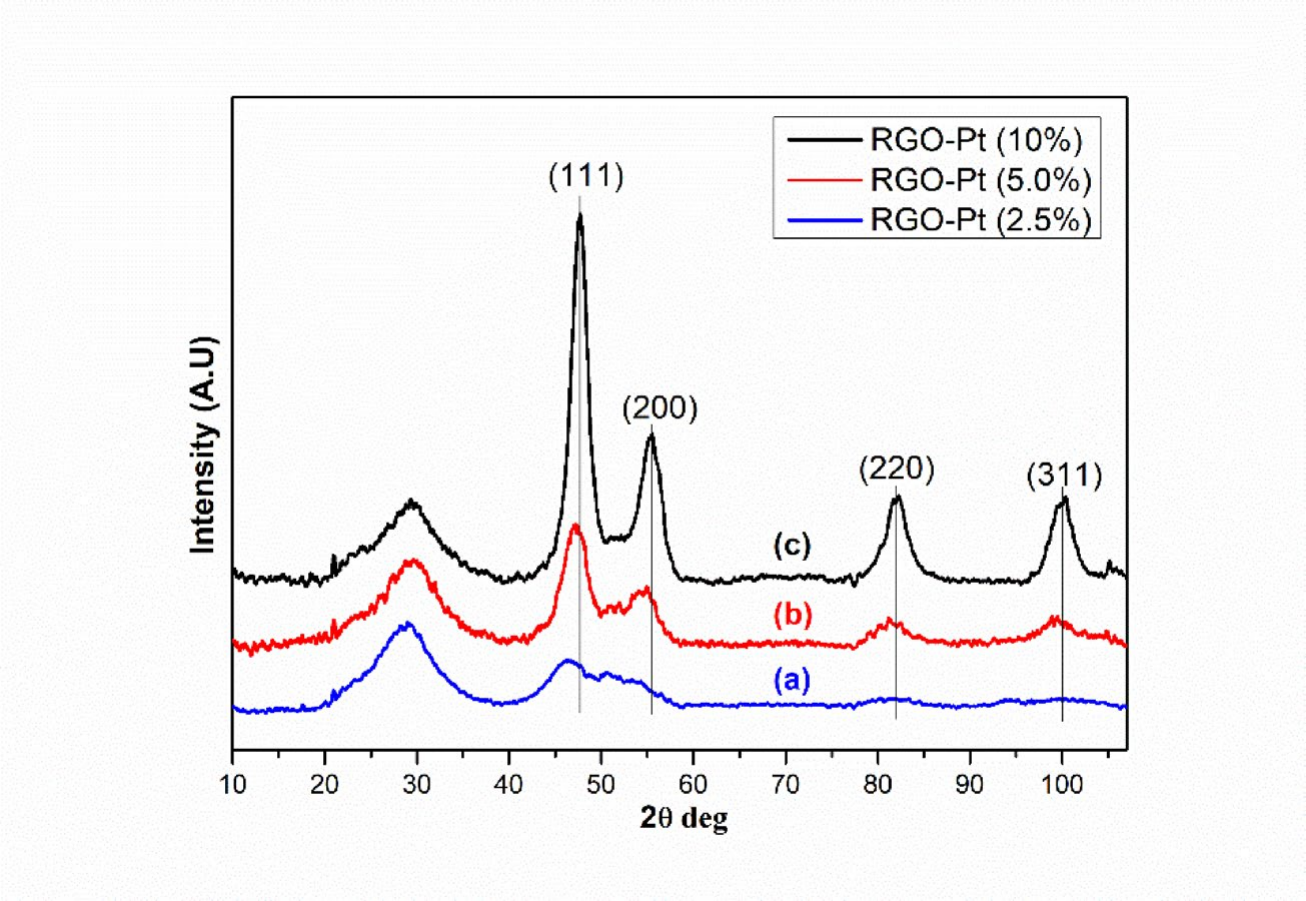
XRD patterns of RGO-Pt (2.5%) (**a**), RGO-Pt (5%) (**b**) and RGO-Pt (10%).

The synthesized GO was initially characterized by XRD, ATR-FTIR and Raman spectroscopy. The XRD pattern of GO (Figure S1) showed a sharp peak at 2θ of 10.91°, a typical characteristic peak of GO with enlarged interplanar distance due to incorporating several oxygen-containing functional groups from oxidation. ATR-FTIR study further confirmed the presence of several oxygen-containing functional groups. In the FTIR spectra of GO (Figure S2), the peak at 1725 cm^−1^ corresponds to the vibrational stretching frequency of C=O. The broad peak between 2200 and 3700 cm^−1^ corresponds to the –OH stretching frequency of alcoholic –OH and carboxylic acid groups present in the GO. The peak at 1217 cm^−1^ corresponds to the C–O–C group present in the system. Raman spectra for GO (Figure S3) also have shown a sharp increase in the intensity ratio (1.29) of the D band and G bands at 1350 cm^−1^ and 1600 cm^−1^, respectively, due to the defects and disorder in the carbon lattice of GO with the incorporation of these oxygen-containing functional groups in GO^22^. Further, the XRD pattern of RGO and Pt-loaded RGO (Figure S1, Fig. 2) shows a broad peak at 27°, a typical characteristic of chemically reduced RGO powder. Apart from this, in the case of Pt-loaded RGO, additional XRD peaks (Fig. 2) at 47.7°, 55.49°, 82.03° and 100.03° correspond to the (111), (200), (220) and (311) planes of Pt nanoparticles present in the sample, respectively (ICCD card # 00-004-0802). The XRD pattern confirms the reduction of the chloroplatinic acid to metallic Pt by ethylene glycol at elevated temperature. The XRD patterns are used to calculate the Pt particle size using the Scherrer formula. The calculated particle size is 1.98 nm, 2.85 nm and 4.24 nm for RGO-Pt with 2.5%, 5% and 10% Pt loading. These results are in good agreement with the particle size distribution calculated from the TEM images of Pt-loaded RGO (discussed later).

**Fig. 3:**
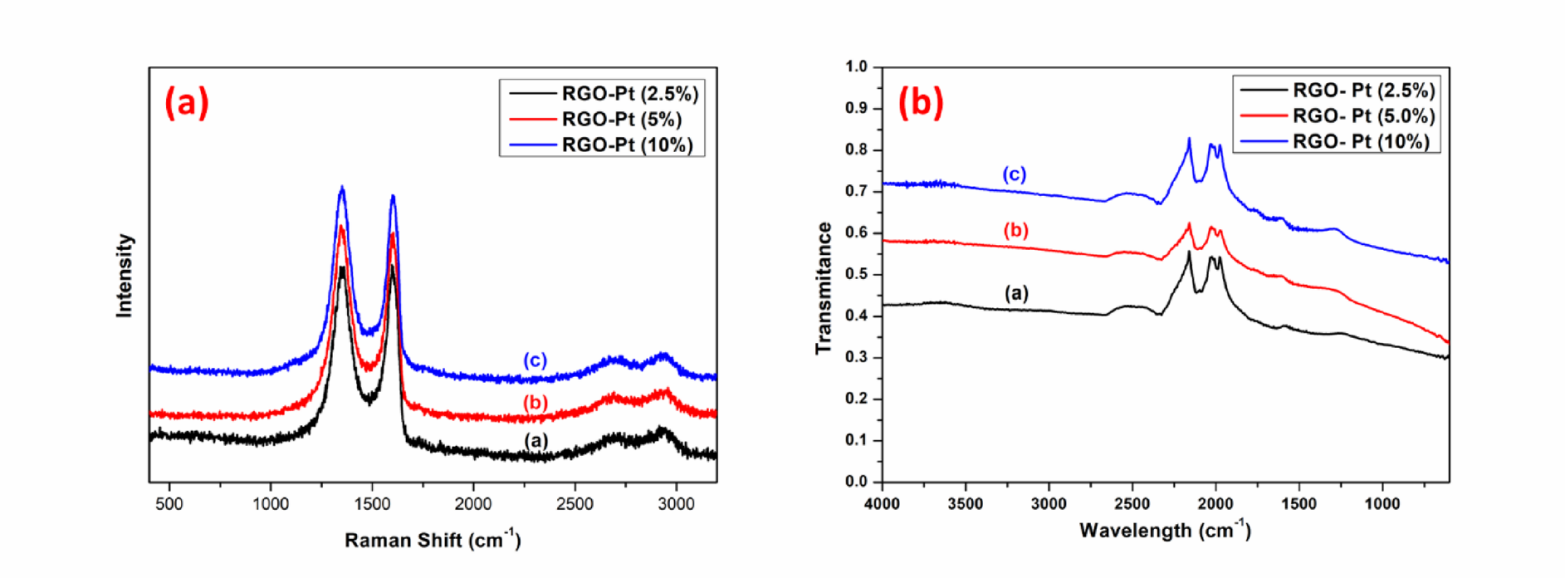
Raman spectra (**a**) of RGO-Pt (2.5%) (**a**), RGO-Pt (5%) (**b**) and RGO-Pt (10%) (**c**). FTIR spectra (**b**) of RGO-Pt (2.5%) (**a**), RGO-Pt (5%) (**b**) and RGO-Pt (10%).

The Raman spectra of the Pt-loaded RGO (Fig. 3a) showed a similar I_D_/I_G_ ratio (1.7 to 1.8) for the D and G bands compared to the RGO, suggesting no additional defect incorporation due to the loading of Pt^23^. However, the ID/IG ratio has increased significantly in all the RGO samples, both unloaded and loaded with Pt, compared to their GO precursor (the I_D_/I_G_ ratio for GO is 1.3).

The increase in the I_D_/I_G_ ratio for RGO samples is attributed to an increase in *sp*^2^ carbon due to the chemical reduction, which alters the structure of precursor GO^23^. A significant decrease in the oxygen-containing functional groups in all the RGO samples was also confirmed from the recorded FTIR spectra (Figure S2, Fig. 3b).

Figure 3 shows the FESEM image of RGO and Pt-loaded RGOs. From the images, it is clearly visible that the graphitic layer structure has been disturbed by the incorporation of Pt nanoparticles. Moreover, with the increase of Pt loading, restacking of the individual RGO sheets has been greatly affected, leading to a decreased interlayer force of attraction. As a result, the dispersibility of the RGO-Pt catalysts in an acidic-aqueous medium also greatly improved with the increase of the Pt content in the catalyst.

**Fig. 4:**
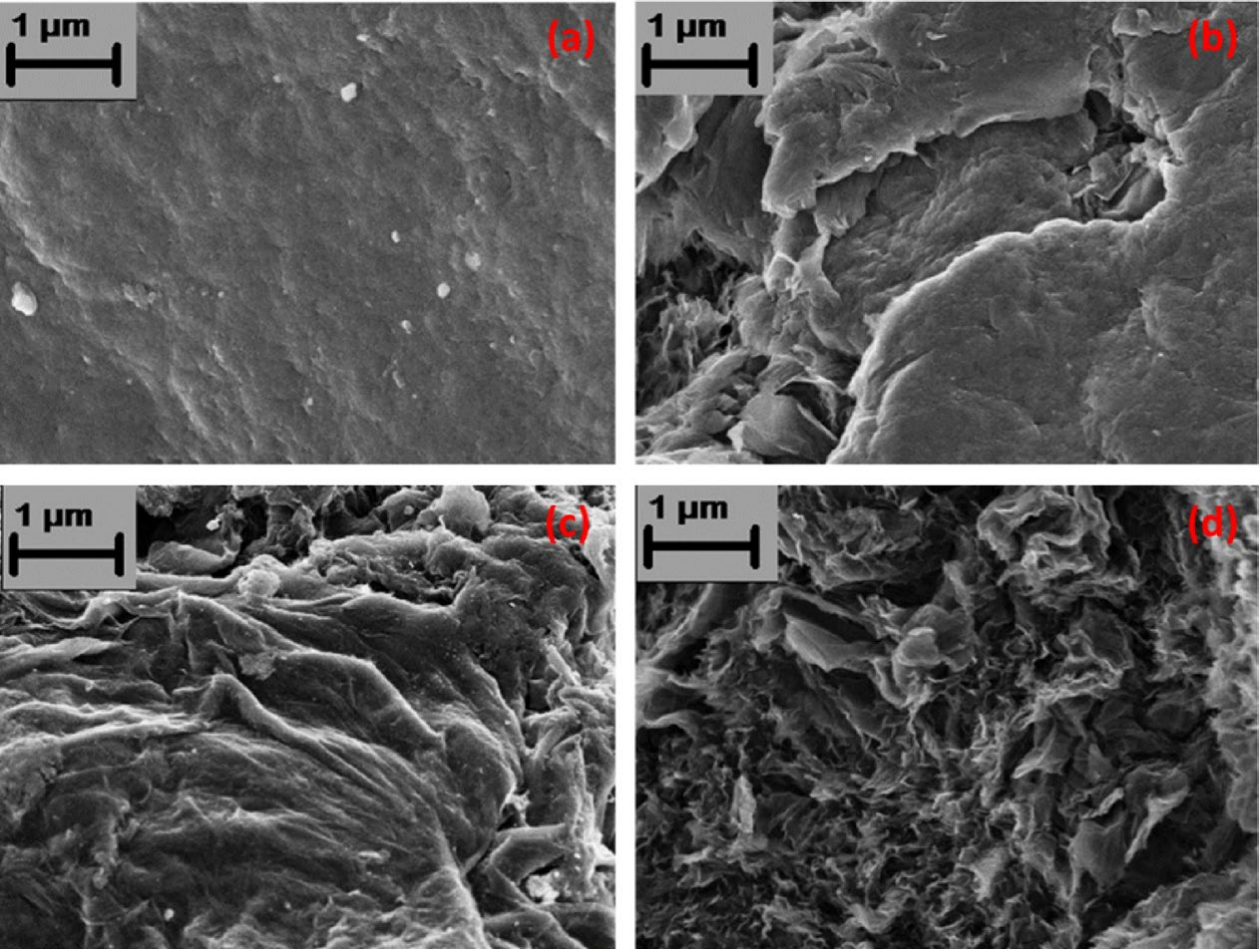
FESEM image of RGO (**a**), RGO-Pt (2.5%) (**b**), RGO-Pt (5%) (**c**) and RGO-Pt (10%) (**d**).

**Fig. 5:**
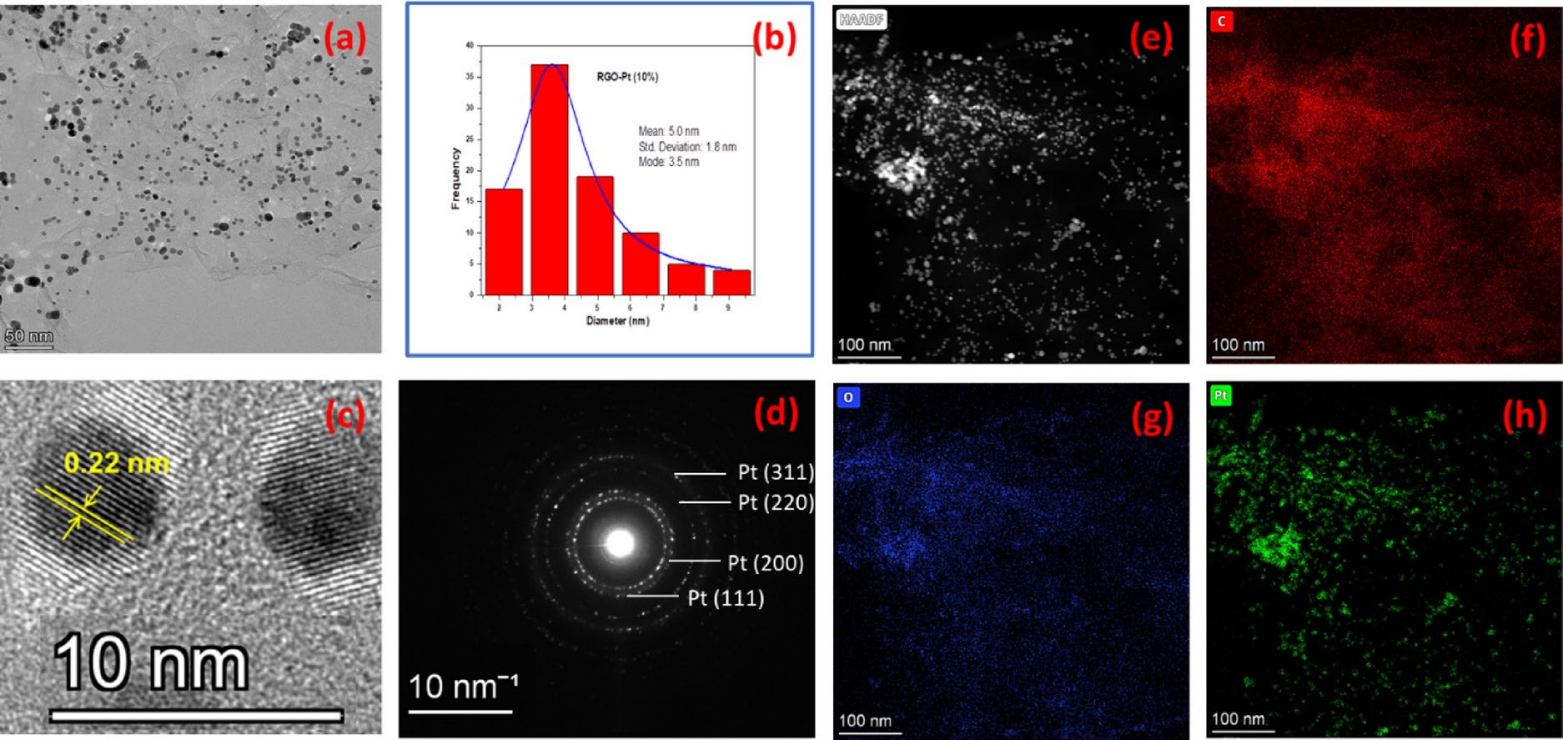
Microstructural characterization of RGO-Pt (10%). TEM image (**a**), Pt particle size distribution (**b**), HRTEM image (**c**), SAED pattern (**d**), STEM-HAADF image (**e**), XEDS elemental map of C (**f**), O (**g**) and Pt (**h**).

The detailed microstructural analysis of RGO-Pt (2.5%) and RGO-Pt (10%) reveals that the individual Pt nanoparticles are dispersed over the RGO sheets in both samples (Figure S4a, Fig. 5a). However, the overall diameter of the Pt nanoparticles has increased significantly, from an average of 1.9 nm in RGO-Pt (2.5%) to an average of 5.0 nm in RGO-Pt (10%) with identical distribution (Figure S4b, Fig. 5b). This result is also consistent with the particle size measured using the Debye–Scherrer formula from the corresponding XRD pattern. The HRTEM images of the dispersed nanoparticles are shown in Figure S6 and Fig. 5c. Fast Fourier Transformation (FFT) analysis of the HRTEM micrographs measures the lattice spacing as 0.22 nm, which corresponds to the (111) plane of the Pt nanocrystal. HRTEM images also confirm that all the Pt nanoparticles that are dispersed over the RGO sheets are single-crystalline in nature, and no polycrystallinity is observed for the Pt nanoparticles in these materials. The HRTEM image of 2.5% Pt-loaded RGO (Figure S6) shows a more ordered structure with an interplanar distance of ~ 0.35 nm for background RGO than that of the 10% Pt-loaded RGO (Fig. 5a), and is consistent with the FESEM images of these materials. The ordered structure of the RGO in the 2.5% Pt-loaded RGO structure is further confirmed from the SAED pattern of the materials (Figure S7), where diffraction rings corresponding to turbostatic layered graphitic carbon material^24^ are observed in comparison to the SAED pattern of 10% Pt-loaded RGO (Fig. 4d), where no such continuous ring is present. The SAED pattern of the 10% Pt-loaded RGO is indexed with the crystallographic planes of Pt. In the HAADF image of RGO-Pt (2.5%) (Figure S7a) and RGO-Pt (10%) (Fig. 5e), Pt particles appear as a bright spot against the dark RGO background due to their high atomic number. Further, the XEDS elemental maps also confirm the bright spots as Pt, whereas the background sheet contains the elements C and O, the constituents of RGO in both cases (Figure S7b–d, Fig. 5f–h).

**Fig. 6:**
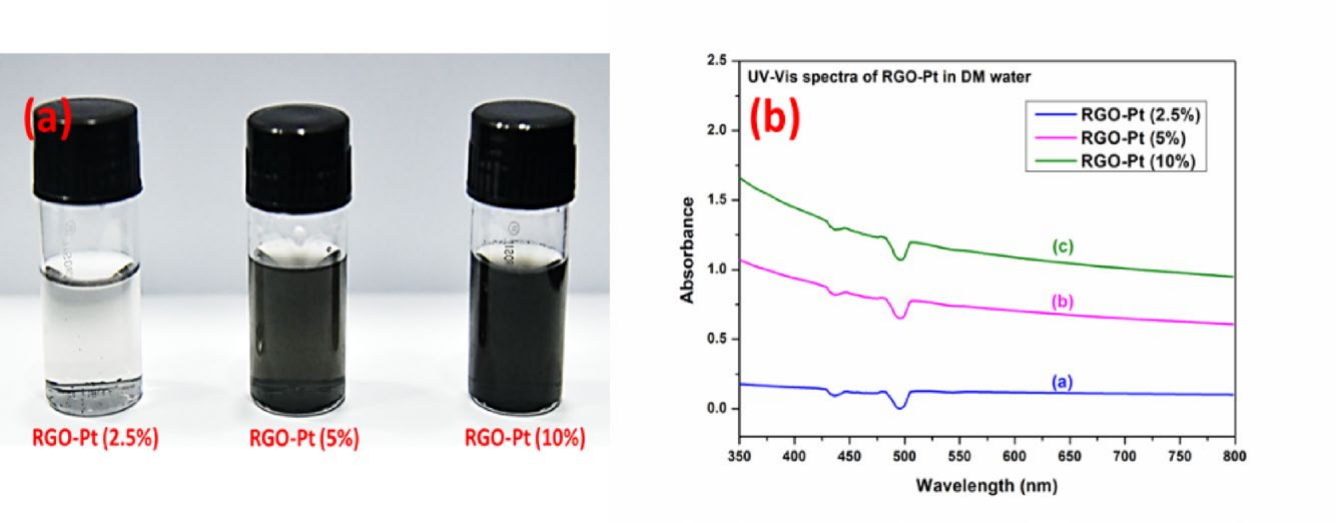
Digital Photographs of RGO-Pt dispersion in water after 3 h of sonication (**a**), UV–Vis spectra of RGO-Pt dispersion (**b**).

To further elucidate the role of Pt in dispersing those materials in water-based solution, a direct experiment was carried out by taking a particular amount of materials (containing the same amount of RGO) in a known volume of water and dispersing by sonication for 3 h. Following this process step, a 3 min incubation period was provided for the dispersion to settle down. After the process, the digital photographs were recorded and are shown in Fig. 6a. The photographs clearly show the increased dispersion of RGO-Pt material with the increase of Pt content. The recorded UV–Vis spectra of the solution also show the increased absorbance for RGO with the increase of Pt content in the RGO-Pt material (Fig. 6b), indicating better dispersion of the material with the increase of Pt content.

The same experiment was carried out in 1.3 M HNO_3_ and 0.2 M N_2_H_4_, and similar findings were also observed (Figure S8).

Further RGO-Pt materials were characterised using XEDS and TOC measurement methods. The results are summarised in Table S1 provided in the supplementary information file. The results show that in all the RGO-Pt samples, the nature of the RGO is quite the same, with increased C content compared to the starting GO. Here, it is also worth noting that the synthetic methods also followed identical conditions synthesizing all the RGO-Pt materials.

### Uranium reduction

When GO was reduced to RGO, chemical reduction removed most of the oxygen-containing functional groups from GO. As a result, the final RGO material becomes hydrophobic, and the dispersion of these materials in the aqueous medium becomes difficult. However, with the loading of Pt, the graphitic structure of the RGO collapsed, helping the material disperse in the acidic aqueous medium, as discussed earlier.

**Fig. 7:**
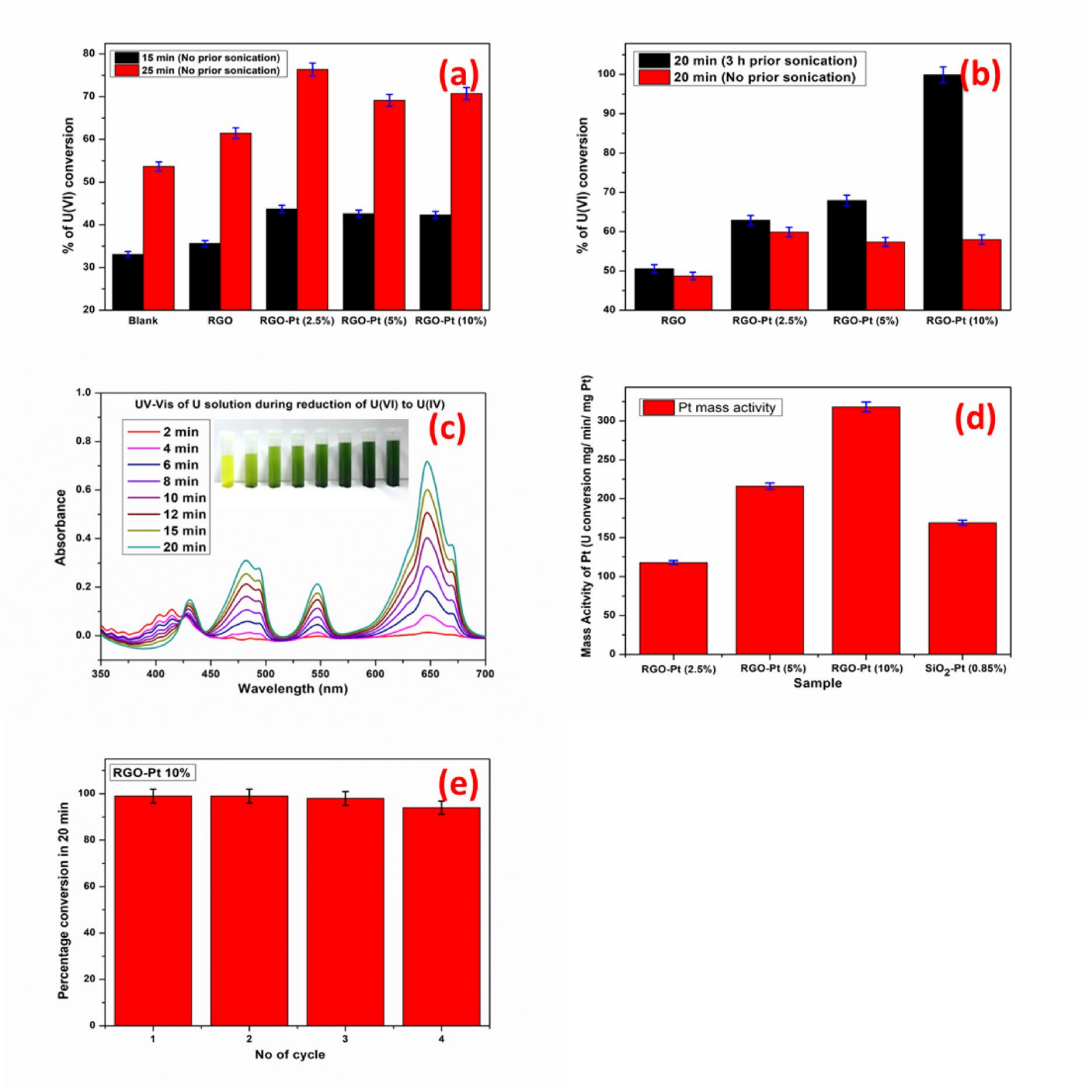
(**a**) Uranium reduction plot with no prior sonication, (**b**) Uranium reduction comparison with and without prior sonication, (**c**) UV–Vis spectra of reaction solution (without catalyst) at different time intervals for RGO-Pt (10%) with prior sonication (inset shows the digital photograph of the solutions). (**d**) Pt mass activity bar plot for RGO-Pt and earlier reported SiO_2_-Pt (0.85%) (**e**) Recyclability bar plot for RGO-Pt (10%).

Two different sets of observations were followed for the uranium reduction reaction with different Pt-loaded RGO. For those experiments where the catalysts were added directly to the uranium solution without any prior dispersion via sonication, the kinetics of the reduction reaction with the 2.5% Pt-loaded RGO catalyst was the fastest. Meanwhile, the intermediate rate of the reaction was observed for the 5% Pt-loaded RGO catalyst, and the 10% Pt-loaded RGO followed the slowest reaction rate (Fig. 7a). The reverse reaction rate was observed with the Pt-loaded RGO catalysts when prior sonication was followed before being subjected to the reduction reaction (Fig. 7b).

In the initial observations, where sonication was not a part of the experiments, a better reaction rate was observed with the 2.5% Pt-loaded RGO due to the smaller Pt particle size in this material. These particles were present at the surface of the agglomerated catalyst and expedited the rate of the reaction. However, with the increase of the Pt loading, the mean Pt particle size increased from 1.9 nm in RGO-Pt (2.5%) to 5.0 nm in RGO-Pt (10%), and the effect was the decreased rate of catalytic U(VI) reduction. Here, it is worth noting that the poor dispersion of these materials in the reaction medium only allowed the limited number of Pt nanoparticles present at the surface of the agglomerated material to participate in the catalytic reduction reaction, and the in-situ stirring with the over-head stirrer was not sufficient to disperse the agglomerates in the reaction medium during the course of the reaction.

However, when prior sonication for 3 h was followed, the 10% Pt-loaded RGO material became well dispersed into the acidic aqueous medium, leaving most of the Pt nanoparticles available on the RGO sheets exposed to the medium to take part in the catalytic U(VI) reduction. Meanwhile, 2.5% Pt-loaded material continued to maintain its agglomerated forms even after continuous sonication for 3 h, leaving most of the Pt nanoparticles embedded into the agglomerates and unavailable for the catalytic reduction reaction. The 5% Pt-loaded RGO followed intermediate dispersibility after 3 h of continuous sonication. This trend in dispersibility is attributed to the disturbance of the graphitic layer structure in these materials with the increase in Pt loading.

The effect of dispersibility was clearly observed in the U(VI) reduction reaction rate. For the 10% Pt-loaded RGO as a catalyst, the reduction reaction rate has improved drastically, and nearly 100% U(VI) to U(IV) conversion was achieved within 20 min from the start of the reaction under the said reaction condition, whereas it was only about 58% without prior sonication. The increase in the rate of the reduction reaction with and without sonication for 2.5% Pt-loaded RGO was marginal (~ 60–63%). In contrast, a slight improvement was observed when 5% Pt-loaded RGO material was used as a catalyst (57.36% to 67.92%) (Fig. 7b).

The experiment performed only with RGO (without any loaded Pt) showed that RGO also positively catalyses the reduction reaction (Fig. 7a), which may be attributed to the semi-metallic/ conducting nature of the RGO sheets. This is a significant improvement made over the earlier reported SiO_2_ matrix^21,25^, where SiO_2_ do not play any role in the U(VI) reduction reaction (Figure S9). Moreover, RGO is a carbonaceous material with little oxygen, so the catalyst matrix is completely incinerable and adds an essential advantage to waste management of the materials used in a radioactive field.

The UV–Vis spectra (Fig. 7c) of the reaction solution are also recorded at different time intervals and show that with time, the absorbance for U(VI) at 414 nm has decreased, with the increase in absorbance for U(IV) at 430 nm, 481 nm, 564 nm and 647 nm. The isosbestic point for this conversion was found at 425 nm, indicating the conversion of U(VI) to U(IV) with the progress of the reaction. At the end of the reaction, no peak at 414 nm was observed, confirming the complete reduction of U(VI) to U(IV).

Figure 7d represents the mass activity of the Pt for the samples with prior dispersion in terms of mg of U converted from U(VI) to U(IV) per minute per mg of Pt employed for the reaction. These data are also compared with the earlier reported^21^ SiO_2_-Pt (0.85%) model catalysts obtained as per the synthetic procedure discussed earlier. The result shows that for RGO-Pt (2.5%), the mass activity of Pt (~ 119 mg/min/mg of Pt) is significantly less than that of the SiO_2_-Pt (0.85%) model catalysts (~ 170 mg/min/mg of Pt). However, for RGO-Pt (5%) and RGO-Pt (10%), the mass activity of the Pt was found to be ~ 216 mg/min/mg of Pt and ~ 319 mg/min/mg of Pt, respectively, indicating the better utilization of Pt in these two samples as compared to the SiO_2_-Pt (0.85%) model catalysts despite the much higher percentage loading of Pt in these two catalysts.

The recyclability of the catalyst was further studied by taking RGO-Pt (10%) as a catalyst of interest. The result of the recyclability study is summarized in Fig. 7e. The use of the catalysts for four consecutive batches of the U(VI) to U(IV) conversion experiment reveals that there is a marginal loss of the catalytic activity for this material in each progressive batch of the experiment, partly attributed to the loss of the catalysts during the recovery of the material.

To understand the stability of RGO-Pt (10%) under the reaction condition, a blank experiment was performed with prior sonication for 3 h in the absence of U (to avoid the dilution effect) for 30 min. After the catalysts were separated, the supernatant was subject to ICP-OES analysis to estimate the Pt concentration in the solution. The Pt estimated in the solution by this method was below the detection limit, suggesting that the catalyst is highly stable in the reaction environment.

Based on our previous studies with SiO_2_/Pt catalysts^21^, we propose RGO-Pt catalyst also undertake a similar mechanism towards reducing U(VI) to U(IV) in HNO_3_-N_2_H_4_ medium since in both the catalysts, Pt acts as an active surface towards catalytic reduction of U(VI) to U(IV) as evident from Fig. 7a. In brief, the mechanism involves the molecular level adsorption of HNO_3_ and U(IV) to the active catalyst surface, followed by interaction with the atomically adsorbed hydrogen over the catalyst, leading to the reduction of U(VI) to U(IV). In this proposed mechanism, the adsorption of HNO_3_ over the surface of the catalyst controls the rate of the reaction. Moreover, the semi-metallic nature of RGO may also positively contribute to the reaction in the case of RGO-Pts. The fact was further verified from the experiments conducted in the presence of no catalysts and in the presence of RGO only.

**Fig. 8:**
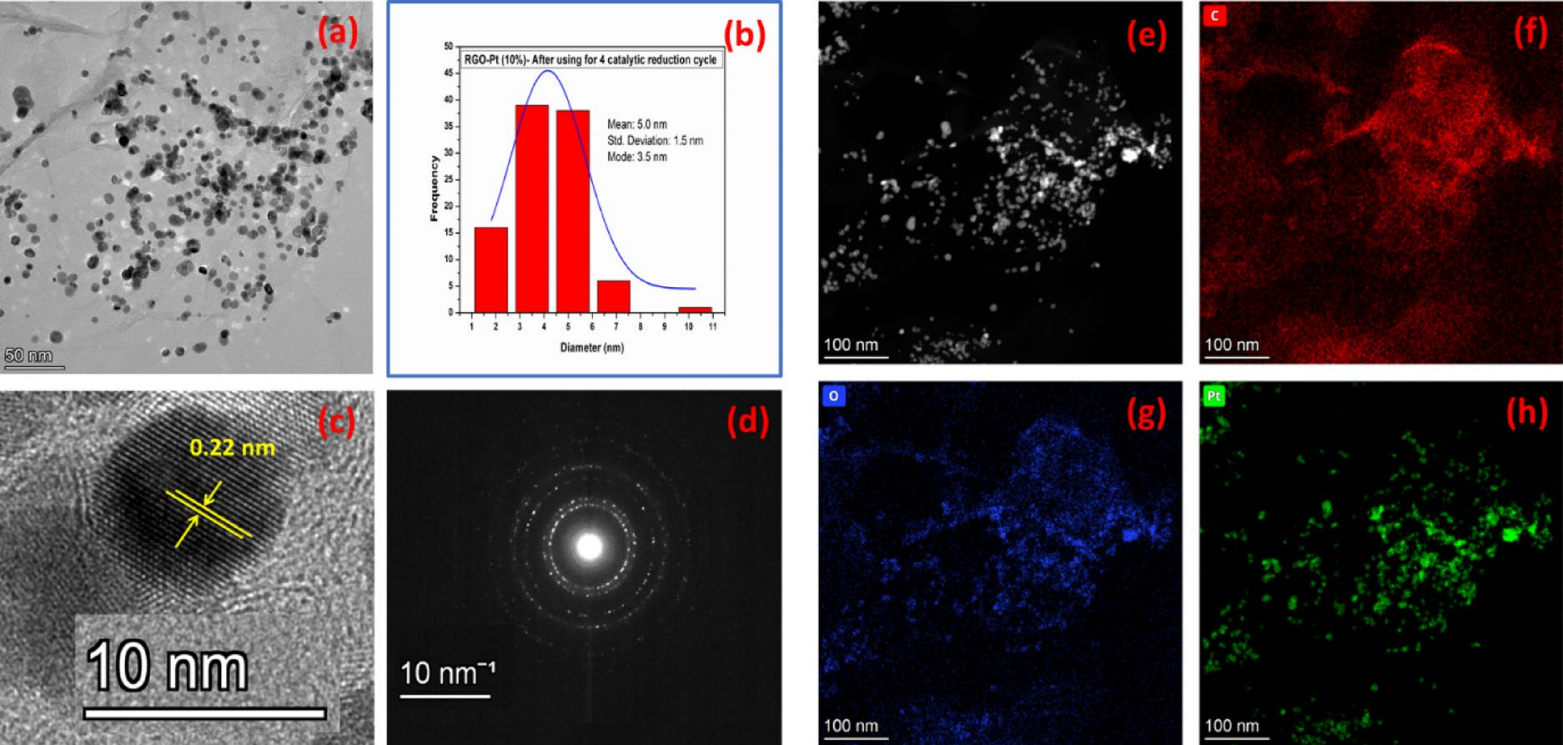
Microstructural characterization of 4 times used RGO-Pt (10%). TEM image (**a**), Pt particle size distribution (**b**), HRTEM image (**c**), SAED pattern (**d**), STEM-HAADF image (**e**) and XEDS elemental map (**f**–**h**).

Further, microstructural analysis of the used catalyst (for 4 consecutive cycles) reveals that the microstructure of the material has remained the same even after use for the four catalytic cycles. Figure 8a shows the TEM image of the used RGO-Pt (10%) with Pt particles distributed over the RGO sheets. The corresponding Pt particle size distribution (Fig. 8b) follows the same pattern as that of the pristine sample, with the mean particle size of 5 nm (same as that of the pristine sample (Fig. 5b)). Other microstructural characterization, like the HRTEM image (Fig. 8c), SAED pattern (Fig. 8d), STEM-HAADF image (Fig. 8e) and XEDS elemental maps (Fig. 8f,h) also contain identical features to those of the pristine sample. The microstructure analysis of the used sample reveals that the RGO-Pt catalysts are highly stable in the reaction environment and may be reused for several cycles as a catalyst for U(VI) to U(IV) conversion.

## Conclusion

In conclusion, Pt-loaded RGO catalysts with Pt loading varied from 2.5 to 10% were synthesized in a safer and ready-to-scale-up method. The dual role of Pt was observed during the application of the catalysts. Pt particles acted as an active site for the U(VI) reduction under the hydrogen atmosphere and helped disperse the Pt-loaded RGO material into the acidic aqueous reaction medium. A 10% Pt-loaded RGO catalyst showed the best performance in this regard. Significantly, RGO also positively catalysed the reduction reaction. The post-application microstructural analysis of the catalyst material confirms that there were no noticeable changes in the microstructure of the catalyst, indicating that the material is very stable under the reaction condition and may be recycled for several batches of reactions without losing the catalytic activity of the material. The synthesized Pt-loaded RGO catalysts were more environmentally benign for ease of waste management with the spent catalysts.

## Electronic supplementary material

Below is the link to the electronic supplementary material.

Supplementary Material 1

## Data Availability

All data are available in the article or additional information file.
