# Supplementary material for: RGO-Pt as an effective catalyst for U(IV) generation under hydrogen
Source: Sci Rep. 2025 Jul 7;15:24218. doi: 10.1038/s41598-025-08442-z (PMC12234972; doi:10.1038/s41598-025-08442-z)
Supplement: Supplementary file 1 — Supplementary Material 1 [file 41598_2025_8442_MOESM1_ESM.docx]

**RGO-Pt as an Effective Catalyst for U(IV) Generation Under Hydrogen**

Kuntal Kumar Pal^1, 2 *^, Ramakrishna Reddy^2, 3^, Chanchal Ghosh^4^, Sandip Dhara^2, 5 *^

^1^ Reprocessing Material Development Section, Process Radiochemistry Reprocessing Research & Development Division, Reprocessing Group, Indira Gandhi Centre for Atomic Research, Kalpakkam- 603102

^2^ Indira Gandhi Centre for Atomic Research, A CI of Homi Bhabha National Institute, Kalpakkam-603102

^3^ Minor Actinide Chemistry and Reconversion Section, Process Radiochemistry Reprocessing Research & Development Division, Reprocessing Group, Indira Gandhi Centre for Atomic Research, Kalpakkam- 603102

^4^ Physical Metallurgy Division, Metallurgy & Materials Group, Indira Gandhi Centre for Atomic Research, Kalpakkam- 603102

^5^ Material Science Group, Indira Gandhi Centre for Atomic Research, Kalpakkam- 603102

Email: [kuntal@igcar.gov.in](mailto:kuntal@igcar.gov.in), [dhara@igcar.gov.in](mailto:dhara@igcar.gov.in)

Table S1. Results of XEDS and TOC analysis

| **Sample** | **XEDS measurement** | | | | **TOC measurement** |
| --- | --- | --- | --- | --- | --- |
|  | **Wt.% (At%) of C** | **Wt.% (At%) of O** | **Wt.% (At%) of Pt** | **At. Ratio of C/O** | **Wt.% of C in RGO**  **(Corrected w.r.t Pt)** |
| GO | 59.30 (66.00) | 40.70 (34.00) | --- | 1.94 | 45.7 (±1.5) |
| RGO | 75.01 (79.99) | 24.99 (20.01) | --- | 3.99 | 76.6 (±3.5) |
| RGO-Pt (2.5%) | 73.83 (79.37) | 25.30 (20.42) | 0.49 (0.03) | 3.88 | 83.9 (±5.9) |
| RGO-Pt (5%) | 73.82 (80.69) | 23.30 (19.12) | 2.87 (0.19) | 4.22 | 81.6 (±2.2) |
| RGO-Pt (10%) | 57.67 (83.60) | 12.63 (13.75) | 29.70 (2.65) | 6.08 | 83.9 (±4.3) |





Figure S1. XRD pattern of GO and RGO





Figure S2. FTIR Spectra of GO and RGO


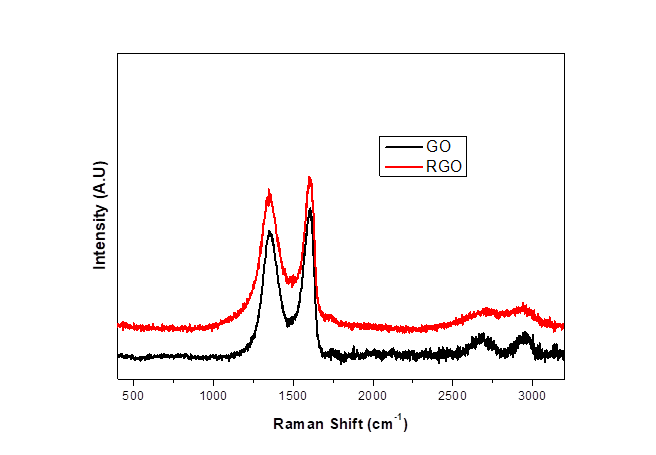


Figure S3. Raman Spectra of GO and RGO


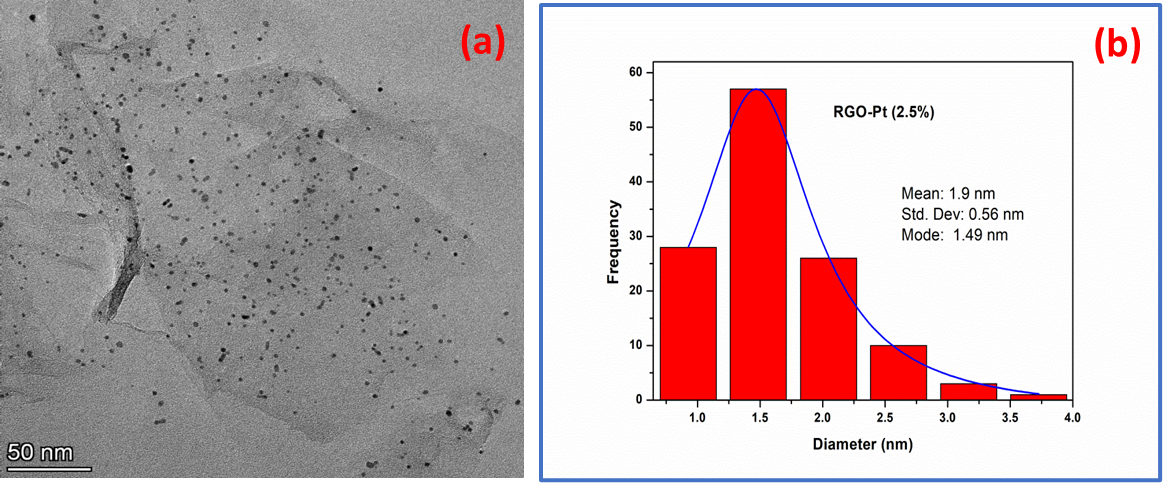


Figure S4. TEM image of RGO-Pt (2.5%) (a) and Pt particle size distribution (b)


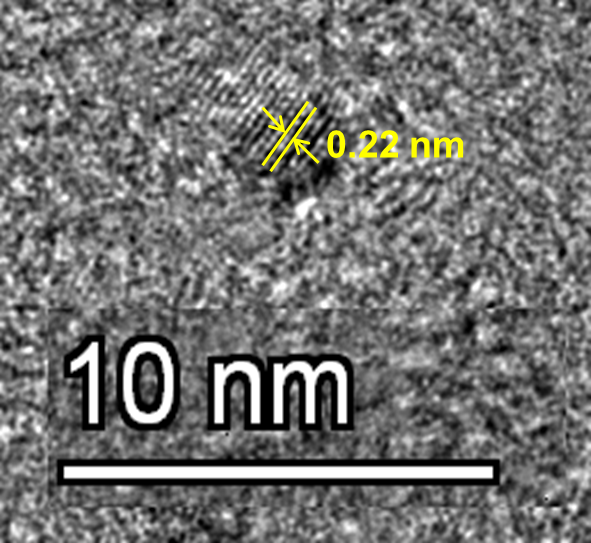


Figure S5. HRTEM image of RGO-Pt (2.5%)


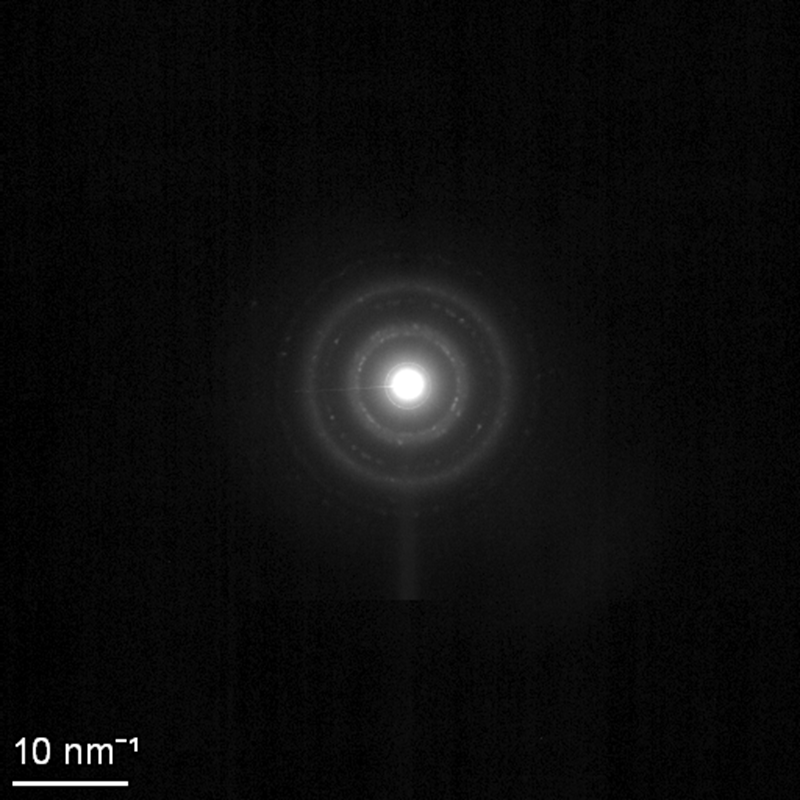


Figure S6. SAED pattern of RGO-Pt (2.5%)


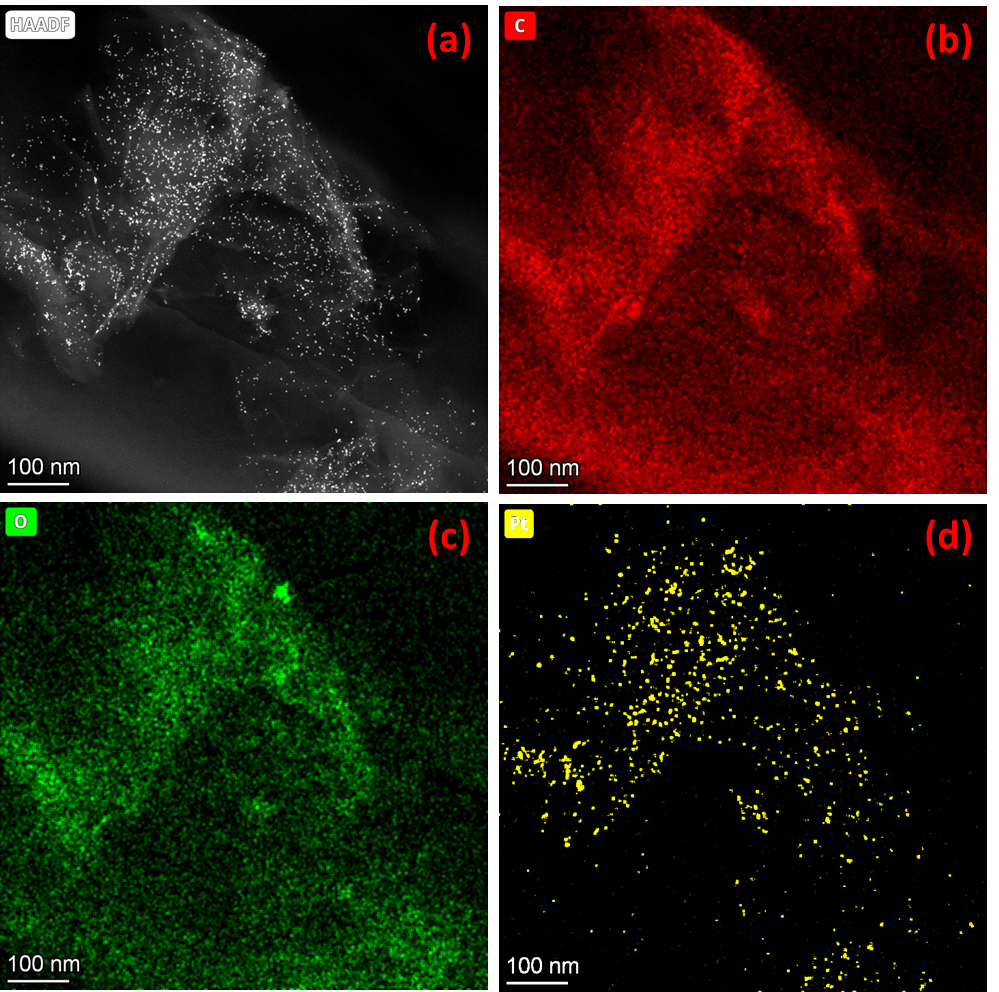


Figure S7. STEM-HAADF image of RGO-Pt (2.5%) (a), XEDS elemental map of C (b), O (c) and Pt (d)


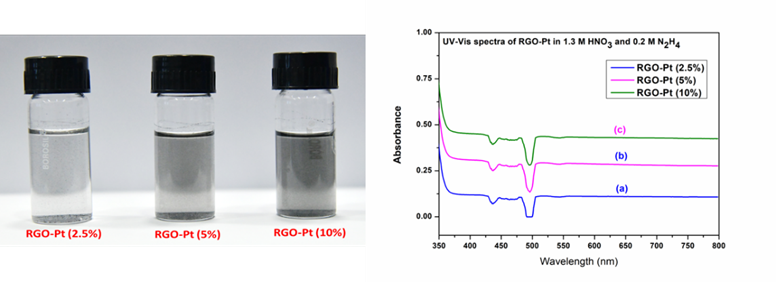


Figure S8. Digital Photographs of RGO-Pt dispersion in HNO_3_-N_2_H_4_ medium after 3 h of sonication (a), UV-Vis spectra of RGO-Pt dispersion in HNO_3_-N_2_H_4_ medium (b)





Figure S9. % U(IV) reduction- comparision between RGO and SiO_2_





Figure S10. Plot of U(IV) concentration (conc.) over time in the absence of a catalyst





Figure S11. Plot of U(IV) concentration (conc.) over time in presence of SiO_2_





Figure S12. Plot of U(IV) concentration (conc.) over time in presence of RGO





Figure S13. Plot of U(IV) concentration (conc.) over time in the presence of RGO-Pt (2.5%) with no prior sonication





Figure S14. Plot of U(IV) concentration (conc.) over time in the presence of RGO-Pt (5%) with no prior sonication





Figure S15. Plot of U(IV) concentration (conc.) over time in the presence of RGO-Pt (10%) with no prior sonication





Figure S16. Plot of U(IV) concentration (conc.) over time in the presence of RGO-Pt (2.5%) with 3 h prior sonication





Figure S17. Plot of U(IV) concentration (conc.) over time in the presence of RGO-Pt (5%) with 3 h prior sonication





Figure S18. Plot of U(IV) concentration (conc.) over time in the presence of RGO-Pt (10%) with 3 h prior sonication
